# Supplementary material for: The ribosome derives the energy to translocate and unwind mRNA from EF-G binding
Source: Nat Commun. 2025 Dec 16;17:121. doi: 10.1038/s41467-025-66812-7 (PMC12775393; doi:10.1038/s41467-025-66812-7)
Supplement: Supplementary file 1 — Supplementary Information [file 41467_2025_66812_MOESM1_ESM.pdf]

Supplementary Information

The Ribosome Derives the Energy to Translocate and Unwind mRNA from EF-G Binding

Hossein Amiri[1,2,3,*,✉], William J. Van Patten[1,2,4,*], Gillian Rexroad[5,6], Varsha P. Desai[1,2], Benjamen A. Sterwerf[1,2], Laura Lancaster[5,6], Harry F. Noller[5,6,✉], Carlos Bustamante[1,2,4,7,8,9,10,✉]

[1]Institute for Quantitative Biosciences-QB3, University of California, Berkeley, CA 94720, USA.
[2]Jason L. Choy Laboratory of Single-Molecule Biophysics, University of California, Berkeley, CA 94720, USA.
[3]Department of Molecular and Cell Biology, University of California, Berkeley, CA 94720, USA.
[4]Biophysics Graduate Group, University of California, Berkeley, CA 94720, USA.
[5]Center for Molecular Biology of RNA, University of California, Santa Cruz, CA 95064, USA.
[6]Department of Molecular, Cell and Developmental Biology, University of California, Santa Cruz, CA 95064, USA.
[7]Department of Chemistry, University of California, Berkeley, CA 94720, USA.
[8]Department of Physics, University of California, Berkeley, CA 94720, USA.
[9]Howard Hughes Medical Institute, University of California, Berkeley, CA 94720, USA.
[10]Kavli Energy Nanoscience Institute, University of California, Berkeley, CA 94720, USA.
* These authors contributed equally
✉ correspondence: mamiri@berkeley.edu (HA), harry@nuvolari.ucsc.edu (HFN), carlosb@berkeley.edu (CB)

This file contains:

Supplementary Figures 1 – 10

Supplementary Tables 1 – 5

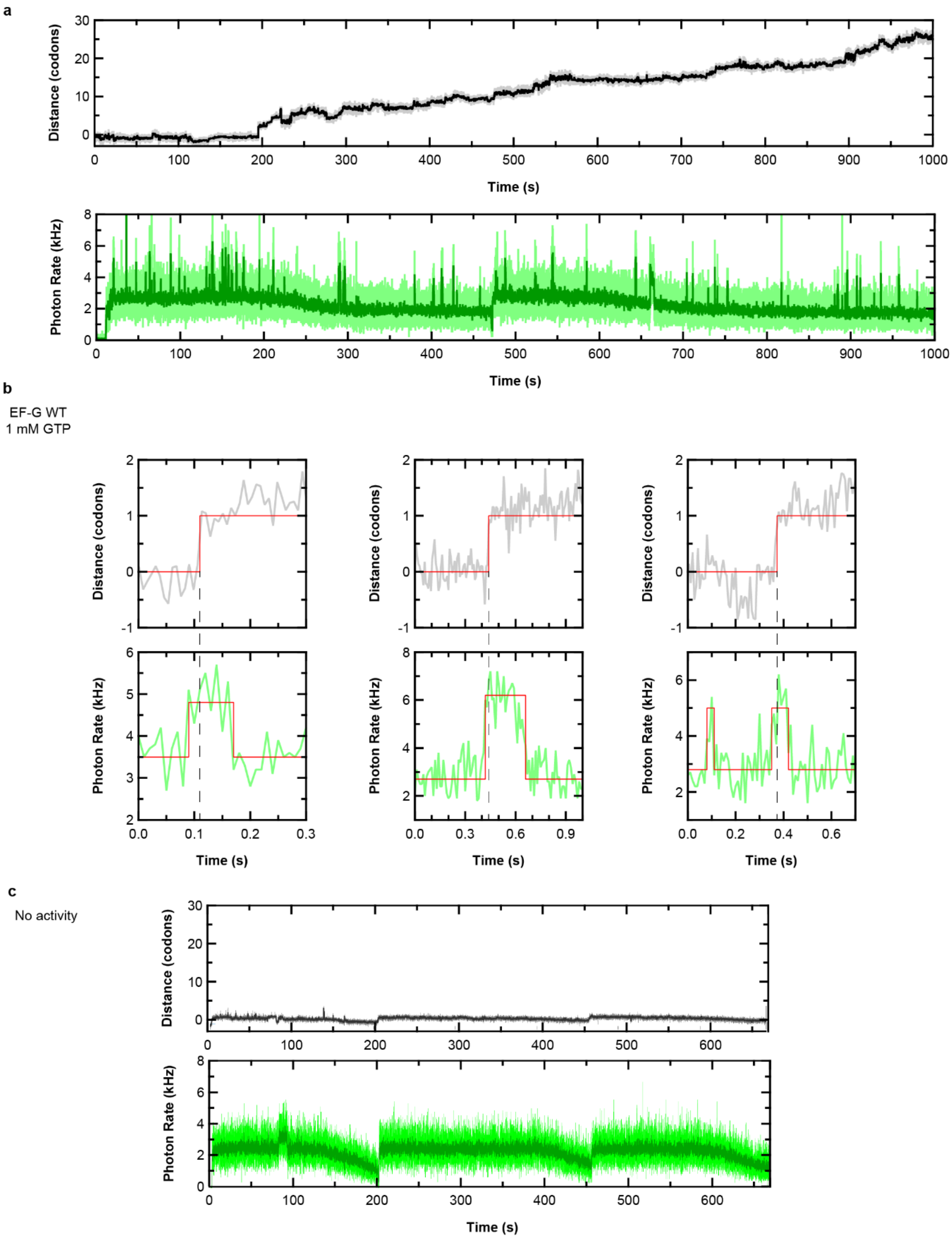
a
Distance (codons)
Time (s)
Photon Rate (kHz)
Time (s)
b
EF-G WT
1 mM GTP
Distance (codons)
Photon Rate (kHz)
Time (s)
c
No activity
Distance (codons)
Photon Rate (kHz)
Time (s)

Supp. Fig. 1| Hairpin unwinding fleezers trajectories for ribosomal translocation with WT EF-G. a, A full representative fleezers trajectory for a single ribosome translocating in the presence of WT EF-G and 1 mM GTP, showing successive unwinding steps (top plot) accompanied by Cy3-labeled EF-G binding events (bottom plot). The raw and 10-point smoothed data are shown in pale and dark colors, respectively, in each plot. Changes in fluorescence baseline are due to mixing of the translation mix in the optical tweezers chamber and opening and closing of the shunt containing translation mix. **b,** Three examples of individual productive WT EF-G binding events. **c,** A fleezers trajectory for a ribosome that does not show activity for over 10 minutes, demonstrating the dependence of unwinding on ribosome activity. Source data are provided as a Source Data file.

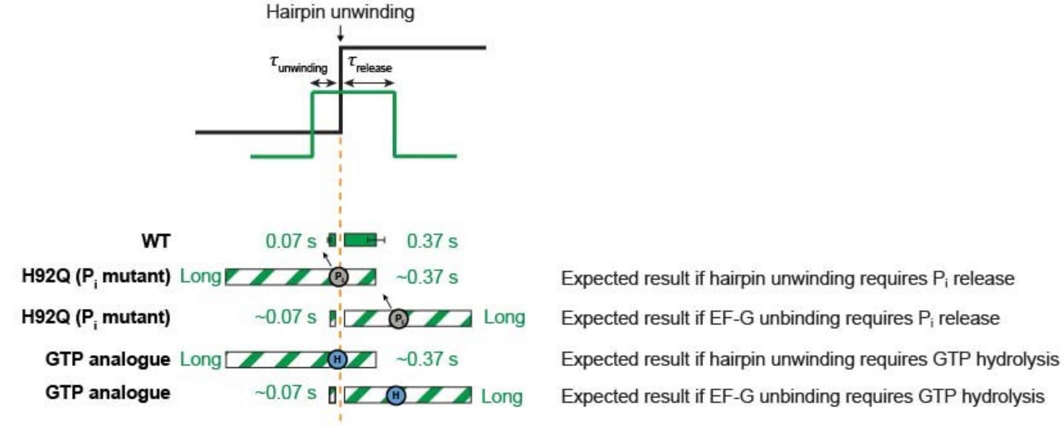

Supp Fig. 2| Possible effects of hindered GTP hydrolysis or P_i release on hairpin unwinding in fleezers trajectories. Lengthening of either $\tau_{unwinding}$ or $\tau_{release}$ is expected if hairpin unwinding or EF-G unbinding, respectively, is affected by the slowed rates in each case. Measured $\tau_{unwinding}$ and $\tau_{release}$ values for WT EF-G are shown for reference.

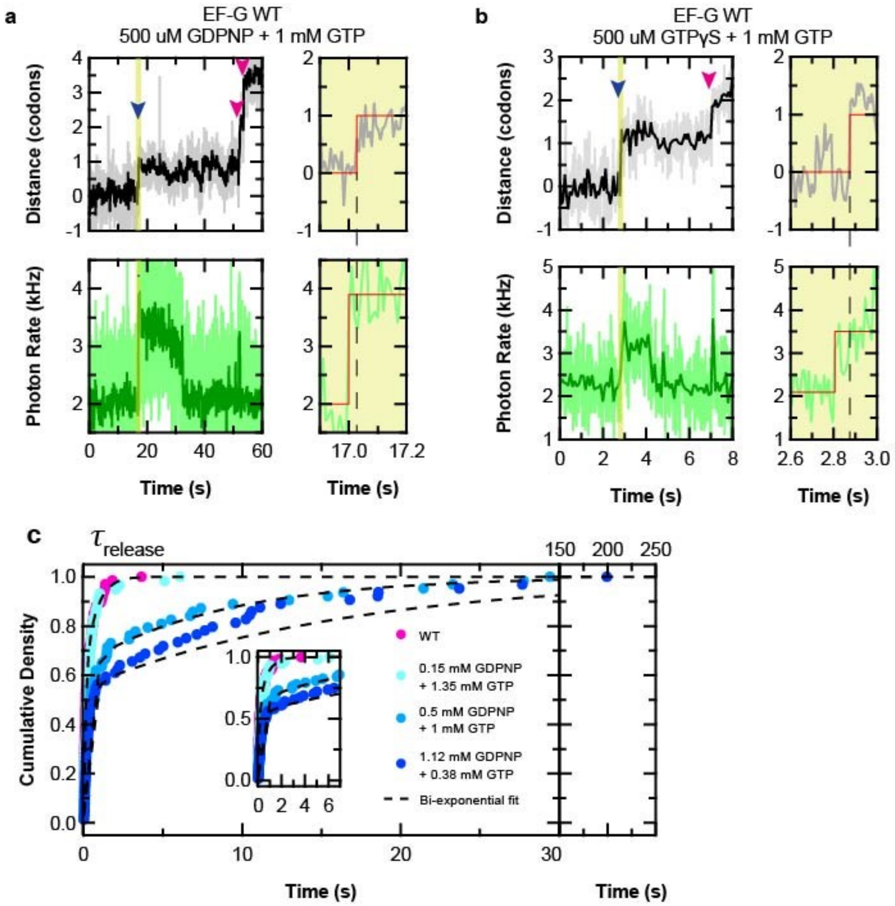

Supp. Fig. 3| Occasional lengthening of $\tau_{release}$ but not $\tau_{unwinding}$ with WT EF-G and a mixture of GTP and GTP analogs. a,b, Examples of productive WT EF-G binding events with a mixture of GTP and GDPNP (a) or a mixture of GTP and GTPγS (b) are shown. In each case, an unwinding step that is associated with a long $\tau_{release}$ (blue arrowhead) is followed by steps (pink arrowhead) with normally short binding events. The time window highlighted in yellow is expanded on the right for each example. **c,** Cumulative distribution plot of $\tau_{release}$ for GTP-only condition (magenta) and three GDPNP:GTP ratios 1:9 (pale blue), 1:2 (medium blue), and 3:1 (dark blue), with bi-exponential fits shown as dashed lines. The time axis is broken between 30 and 150 seconds due to a lack of data points. Source data are provided as a Source Data file.

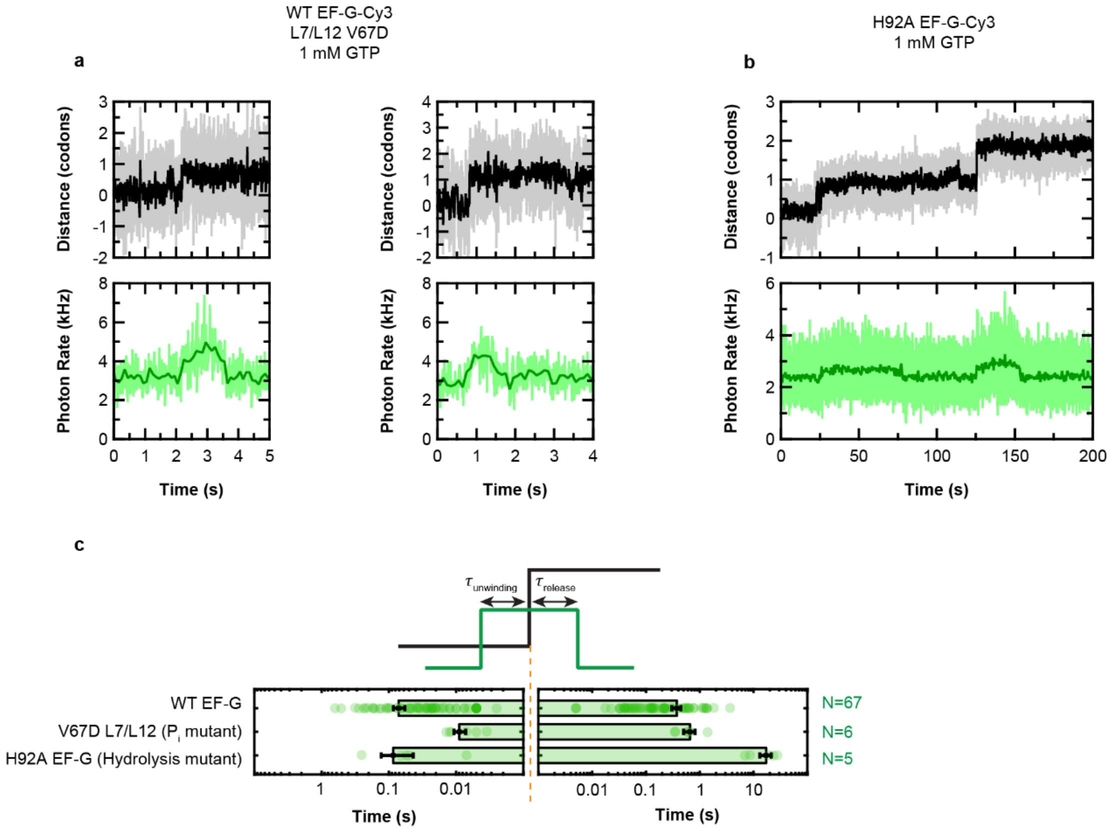

Supp. Fig. 4| Lengthening of $\tau_{release}$ but not $\tau_{unwinding}$ with ribosomal mutant V67D L7/12 (slow P_i release) or with EF-G mutant H92A (slow GTP hydrolysis and P_i release). a, Examples of productive WT EF-G binding events in the presence of ribosomes containing mutant V67D L7/L12. **b**, Examples of productive hydrolysis mutant (H92A) EF-G in the presence of WT ribosomes. **c**, Summary of $\tau_{unwinding}$ and $\tau_{release}$ measurements for V67D L7/L12 and H92A EF-G, represented as mean ± standard error. Individual data points are shown as small circles, and the number of data points (N) is indicated on the right. Source data are provided as a Source Data file.

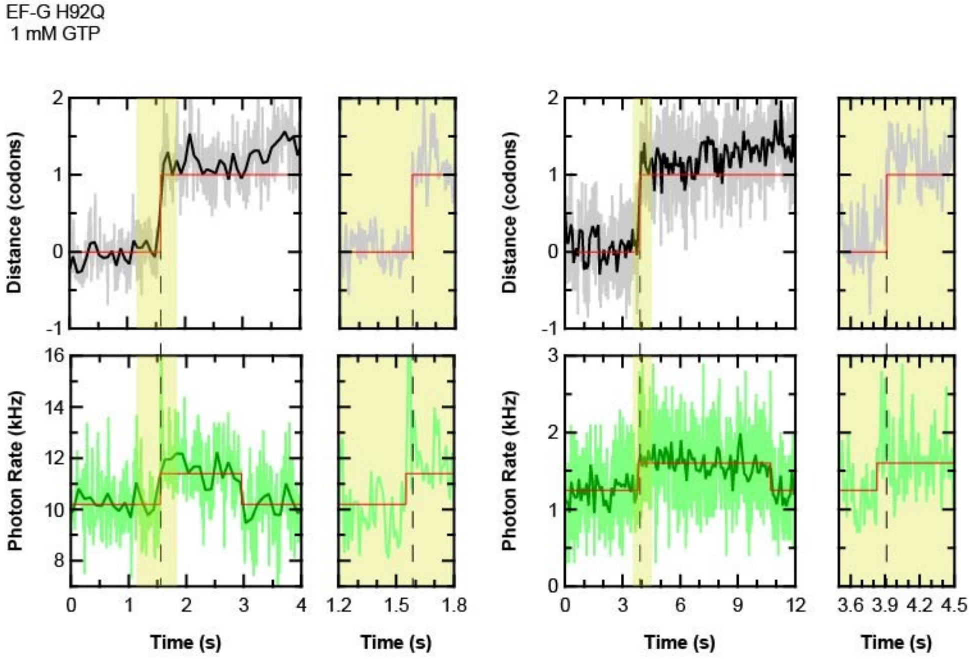

Supp. Fig. 5| Lengthening of $\tau_{release}$ but not $\tau_{unwinding}$ with EF-G mutant H92Q (slow P_i release). Two examples of productive H92Q EF-G binding events are shown. The time window highlighted in yellow is expanded on the right for each example to better resolve the short time between EF-G binding and the hairpin unwinding step. Source data are provided as a Source Data file.

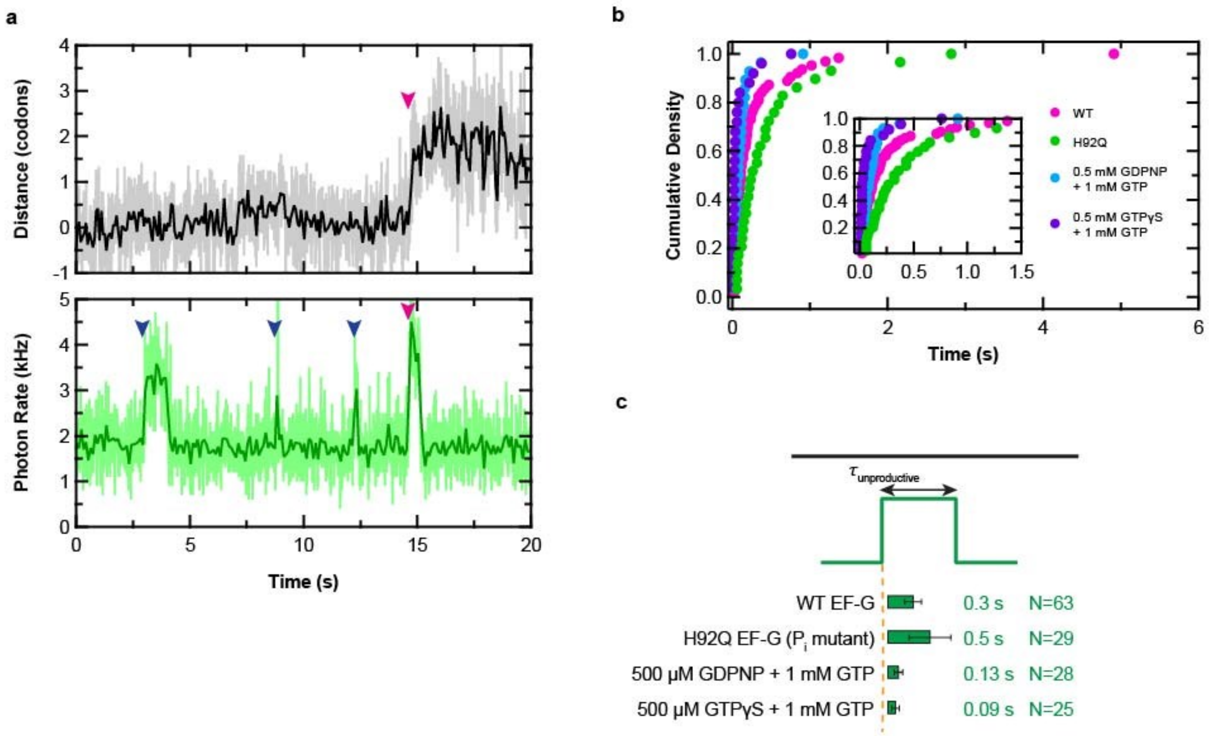

Supp. Fig. 6| Insensitivity of unproductive EF-G binding events to GTP hydrolysis or P_i release. a, A fleezers trajectory showing multiple unproductive EF-G binding events (blue arrowheads) before a productive EF-G binding event accompanied by an unwinding step (pink arrowheads). **b,** A cumulative distribution plot for $\tau_{unproductive}$ under various conditions. The inset in the plot shows a magnified view for the shorter observed times. **c**, Summary of $\tau_{unproductive}$ measurements for WT EF-G, H92Q EF-G, the GDPNP mixture, and the GTPγS mixture, represented as mean ± standard error. The number of data points (N) is indicated for each condition. Source data are provided as a Source Data file.

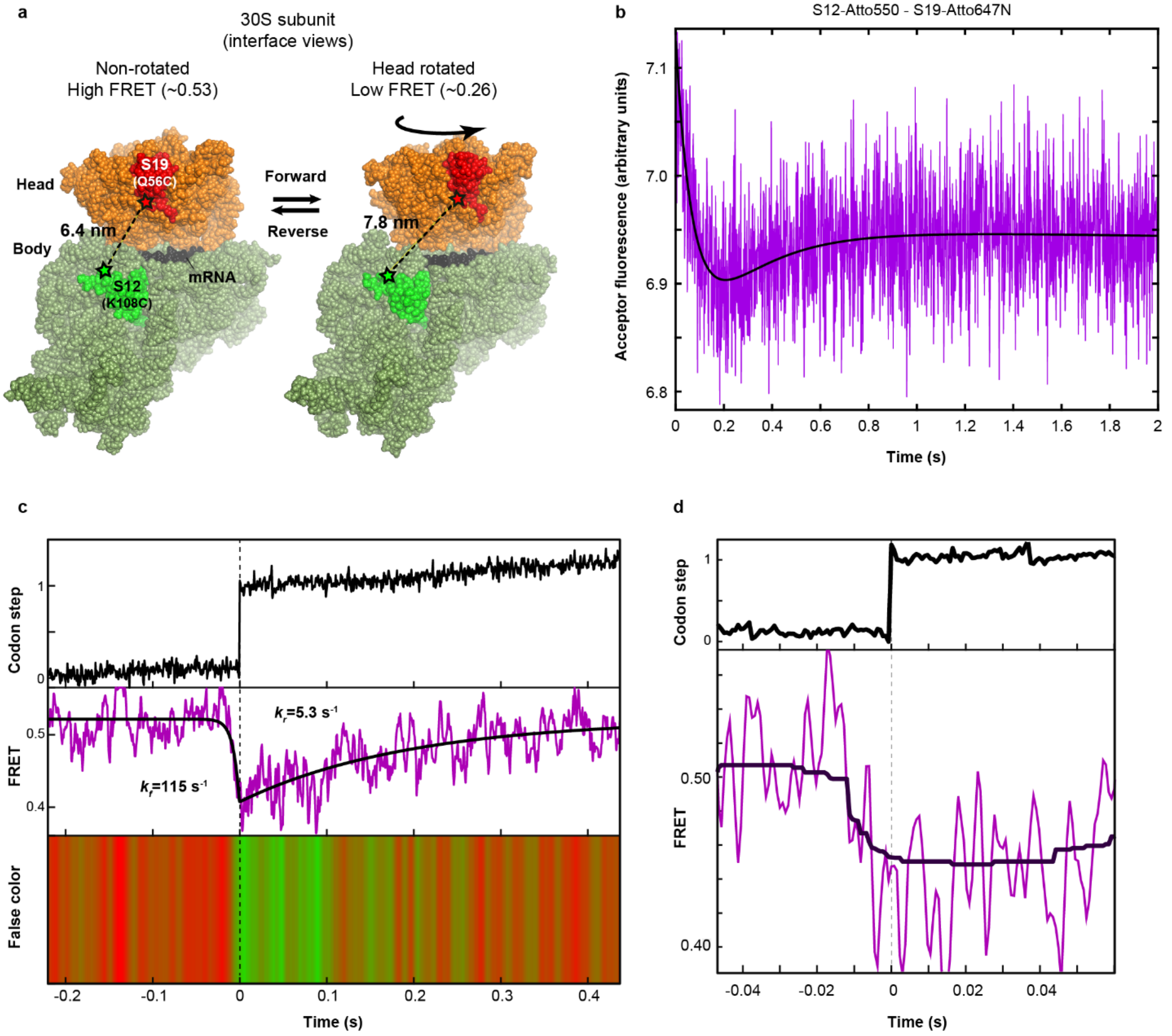
a
30S subunit
(interface views)
Non-rotated
High FRET (~0.53)
Head rotated
Low FRET (~0.26)
Head
Body
S19
(Q56C)
S12
(K108C)
mRNA
6.4 nm
7.8 nm
Forward
Reverse
b
S12-Atto550 - S19-Atto647N
Acceptor fluorescence (arbitrary units)
Time (s)
c
Codon step
FRET
False color
k_r=115 s^{-1}
k_r=5.3 s^{-1}
Time (s)
d
Codon step
FRET
Time (s)

Supp. Fig. 7| FRET measurements of double-labeled ribosomes during translocation. a, Labeling sites of Atto550 (donor) and Atto647N (acceptor) on cysteines introduces in proteins S12 (body domain) and S19 (head domain) respectively[37], and their distances before and after head rotation observed during translocation (PDB: 7SSL, 7SS9 [22]). An increase in distance (and decrease in FRET) is expected upon head rotation. The predicted FRET values 0.53 and 0.26 (FRET change of 0.27) are calculated based on a Förster radius (R_0) of 6.5 nm for the Atto550-Atto647N pair. **b,** Result of stopped-flow kinetic measurement of single-turnover translocation after addition of EF-G and GTP to double-labeled pre-translocation complexes. Acceptor fluorescence is plotted as a function of time and fitted to a biphasic function, showing an initial decrease followed by an increase in fluorescence, corresponding to forward and reverse 30S head domain rotations, respectively[24,37]. **c,** Event averaging from the multi-turnover single-molecule hairpin unwinding assay with double-labeled ribosomes showing a rapid average drop in FRET around the unwinding time followed by a slow recovery. A smaller low-pass filter window (15 ms) is used here compared to Fig. 2d (68 ms) to better show the rapid FRET drop. The two-sided exponential fit and extracted rate parameters are shown. The fitting places the center (time of lowest FRET) close to the unwinding time (±4 ms). **d,** Magnified view of unfiltered (purple) and median-filtered (dark purple) FRET average showing FRET decrease beginning slightly prior to unwinding and reaching a minimum a few milliseconds after unwinding. Source data are provided as a Source Data file.

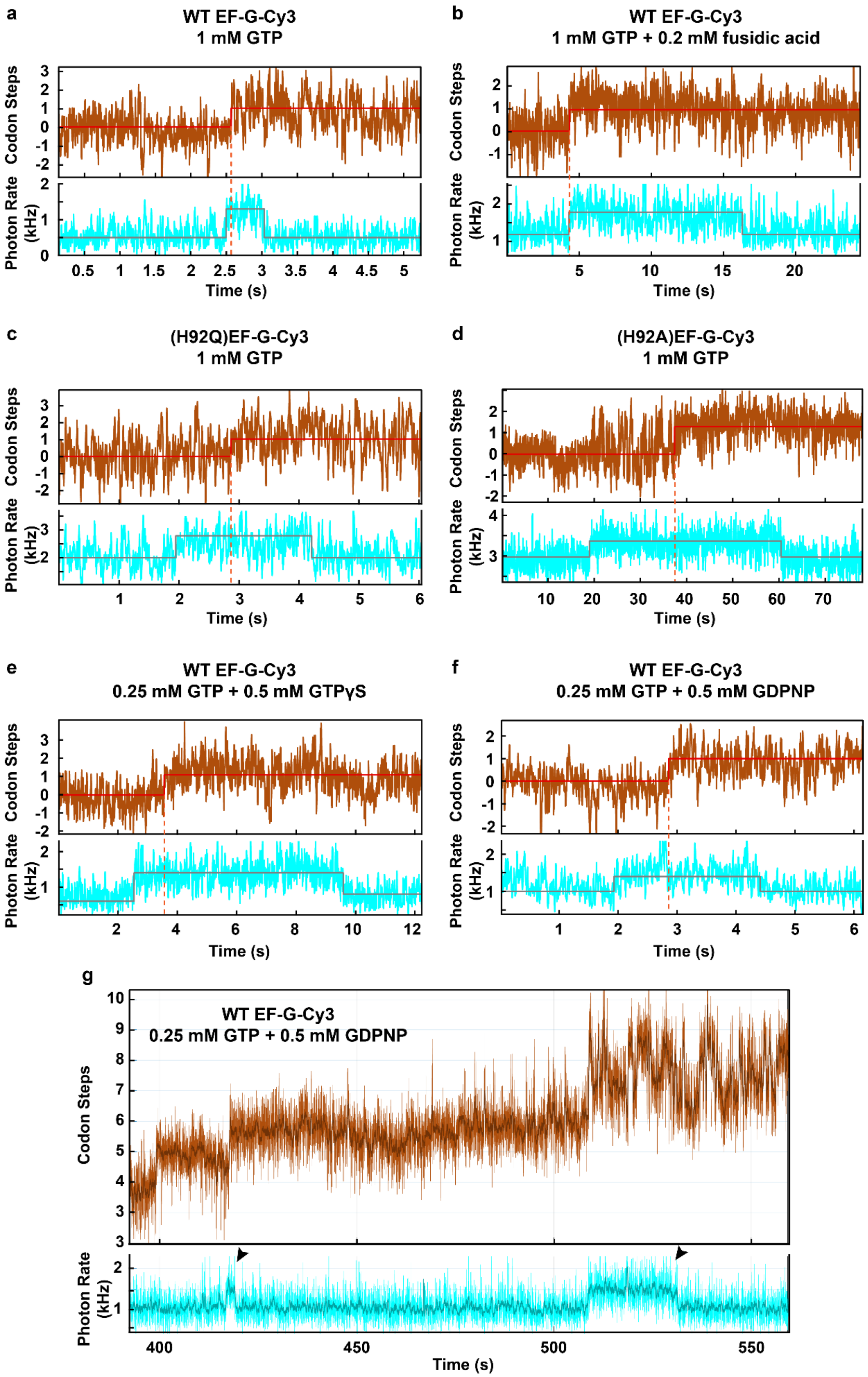
a
WT EF-G-Cy3
1 mM GTP
Codon Steps
Photon Rate (kHz)
Time (s)
b
WT EF-G-Cy3
1 mM GTP + 0.2 mM fusidic acid
Codon Steps
Photon Rate (kHz)
Time (s)
c
(H92Q)EF-G-Cy3
1 mM GTP
Codon Steps
Photon Rate (kHz)
Time (s)
d
(H92A)EF-G-Cy3
1 mM GTP
Codon Steps
Photon Rate (kHz)
Time (s)
e
WT EF-G-Cy3
0.25 mM GTP + 0.5 mM GTPγS
Codon Steps
Photon Rate (kHz)
Time (s)
f
WT EF-G-Cy3
0.25 mM GTP + 0.5 mM GDPNP
Codon Steps
Photon Rate (kHz)
Time (s)
g
WT EF-G-Cy3
0.25 mM GTP + 0.5 mM GDPNP
Codon Steps
Photon Rate (kHz)
Time (s)

Supp. Fig. 8| Fleezers trajectories from the assisting force assay. a-f, Examples of productive EF-G binding events in the assisting force assay (in the presence of biotinylated ribosomes) are shown for the conditions indicated. Note that the time scales of the graphs for different conditions are not the same, and data are down-sampled accordingly for clarity. **g,** A translocation trajectory in the presence of GDPNP, showing two long binding events (arrowheads). The raw and 10-point smoothed data are shown in pale and dark colors, respectively. Source data are provided as a Source Data file.

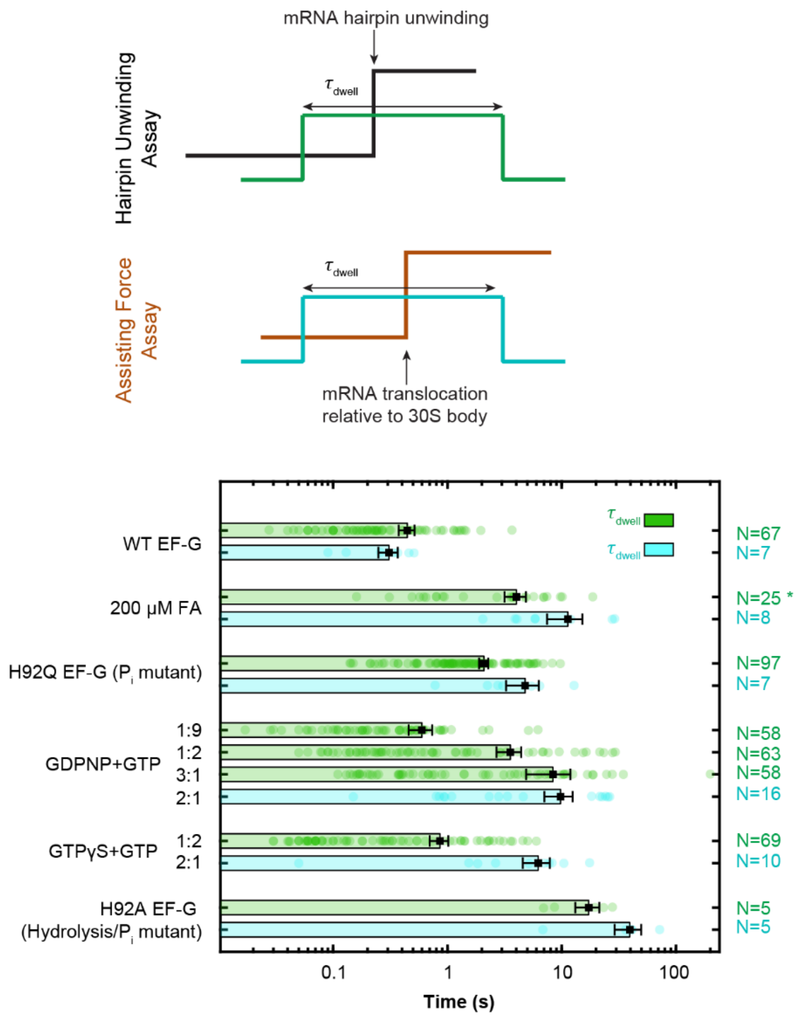

Supp. Fig. 9| Total EF-G dwell times in the hairpin unwinding and assisting force assays. Summary of total Cy3-labeled EF-G dwell times on the ribosome for productive steps in the hairpin unwinding (green) and assisting force (cyan) assays, represented as mean ± standard error. Under perturbed conditions, the total EF-G dwell times in the assisting force assay are longer than those in the hairpin unwinding assay. The small sample sizes for the assisting force assay (N, indicated on the right), or differences in the experimental setup could have caused this apparent difference. In particular, for GDPNP and GTPγS, a higher analog:GTP ratio was used in the assisting force assay (2:1) compared to the hairpin unwinding assay (1:2), which, as expected, contributed to relatively longer total dwell times in the assisting force assay. Nevertheless, the effects of perturbations when compared to the unperturbed condition (WT EF-G with GTP) are qualitatively similar in both assays. The hairpin assay data with fusidic acid (FA, *) were obtained previously[32]. Source data are provided as a Source Data file.

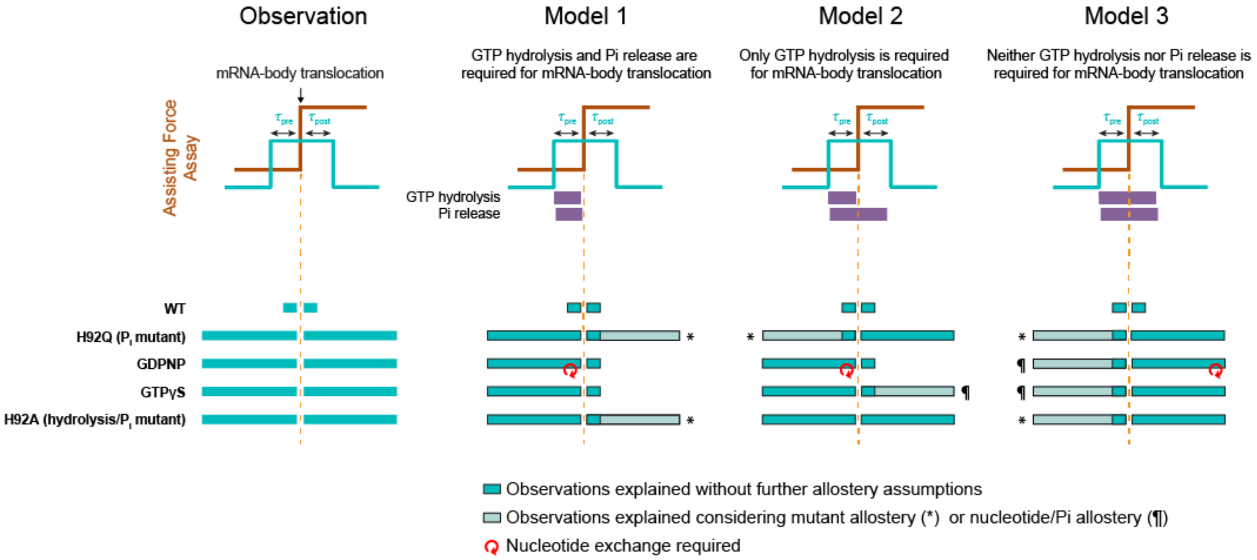

Supp. Fig. 10| Models for the requirement of GTP hydrolysis and P_i release for mRNA translocation relative to the 30S body domain. Three models can be imagined for the requirement for GTP hydrolysis and P_i release in mRNA-body translocation. Note that the models are concerned with the time by which these chemical steps are thermodynamically required, not the time that they normally occur (which can be earlier). The range of occurrence time is indicated by the purple color bars. The observations from the assisting force assay are displayed qualitatively on the left, showing that both τ_{pre} and τ_{post} are lengthened when EF-G mutant or GTP analogs are used compared to unperturbed (WT) conditions. For each model, the predictions for the listed conditions without considering kinetic effects are shown in dark cyan, with pale cyan indicating augmented predictions when kinetic effects on mRNA-body translocation due to the mutants (*) or analogs (¶) are also considered. What we mean by a kinetic effect of GTP hydrolysis (or P_i release) is that these reactions accelerate mRNA-body translocation while not being strictly and thermodynamically required for it, which is equivalent to stating that GTP hydrolysis (or P_i release) lowers the activation energy of translocation without being needed to provide the free energy to drive translocation. In model 3, a kinetic effect of GTP hydrolysis on mRNA-body translocation can explain the observed lengthening of τ_{pre} by the GTP analogs and the H92A mutant (and also the H92Q mutant if it has some hydrolysis deficiency in addition to its reported P_i release deficiency). Similarly, in model 2, a kinetic effect of P_i release on mRNA-body translocation can explain the lengthening of τ_{pre} by the H92Q mutant. The normal acceleration of mRNA-body translocation by canonical GTPase activity, or conversely the translocation slowdown due to GTP analogs or EF-G mutations, must occur via an allosteric mechanism that communicates the subtle change in the local environment of the EF-G nucleotide binding site to the ribosome complex. Importantly, mutations or GTP analogs could allosterically slow down conformational changes required for mRNA-body translocation or EF-G dissociation (thus lengthening τ_{pre} or τ_{post}, respectively) regardless of their GTP hydrolysis and/or P_i release deficiencies *per se*. For instance, H92A and H92Q EF-G mutants may inherently be slow in supporting mRNA-body translocation (explaining the long τ_{pre} in models 2 and 3) or in dissociation from the ribosome (long τ_{post} in model 1), aside from

their slow GTPase activities. Similarly, the thiophosphate residue after the slow hydrolysis of GTPγS may itself be also slow to release from EF-G when compared to the canonical P_i (long τ_{post} in model 2). The red circle arrow in the GDPNP row marks the time by which GTP exchange must occur in order to avoid a practically infinite lengthening. Only the predictions from model 3 match the observations. In both models 1 and 2, GDPNP exchange to GTP must occur prior to mRNA-body translocation, after which there is no reason for an abnormally long τ_{post}. Additionally, in model 1, after GTPγS (slow) hydrolysis and thiophosphate release, the system is in a WT-like state and a long τ_{post} afterwards is not readily explainable.

Supp. Table 1| MLE fitting results for a double exponential distribution describing $\tau_{unwinding}$. Errors are reported as two standard deviations (95%) confidence intervals of the fits. Fitting $\tau_{unwinding}$ to a single exponential distribution for any condition always yielded a poor fit. The last column shows sample mean and standard error of the mean (SEM).

$\tau_{unwinding}$

	f_1	k_1	k_2	N event	N ribosome	Mean ± SEM
WT	39 ± 24%	6.7 ± 3.7 s^{-1}	45 ± 25 s^{-1}	67	24	70 ± 14 ms
H92Q	24 ± 16%	3.3 ± 1.9 s^{-1}	29 ± 11 s^{-1}	97	29	100 ± 20 ms
GDPNP+GTP (1.12+0.38 mM)	18 ± 16%	1.5 ± 1.2 s^{-1}	30 ± 17 s^{-1}	58	40	160 ± 60 ms
GDPNP+GTP (0.5+1 mM)	64 ± 25%	14 ± 6 s^{-1}	95 ± 75 s^{-1}	63	27	50 ± 8 ms
GDPNP+GTP (0.15+1.35 mM)	21 ± 20%	5 ± 4 s^{-1}	44 ± 22 s^{-1}	58	23	60 ± 15 ms
GTPγS+GTP (0.5+1 mM)	22 ± 27%	11 ± 9 s^{-1}	51 ± 24 s^{-1}	69	20	35 ± 7 ms

Supp. Table 2| MLE fitting results for $\tau_{release}$. Errors are reported as two standard deviations (95%) confidence intervals of the fits. A single-exponential fit was performed if sufficient. The last column shows sample mean and standard error of the mean (SEM).

$\tau_{release}$

	k_{single}	f_1	k_1	k_2	N event	N ribosome	Mean ± SEM
WT	-	45 ± 27%	1.4 ± 0.7 s^{-1}	9 ± 6 s^{-1}	67	24	374 ± 70 ms
H92Q	0.5 ± 0.1 s^{-1}	-	-	-	97	29	2 ± 0.2 s
GDPNP+GTP (1.12+0.38 mM)	-	45 ± 13%	0.06 ± 0.02 s^{-1}	3.4 ± 1.5 s^{-1}	58	40	8.2 ± 3.4 s
GDPNP+GTP (0.5+1 mM)	-	35 ± 15%	0.11 ± 0.05 s^{-1}	2.8 ± 1.4 s^{-1}	63	27	3.5 ± 0.9 s
GDPNP+GTP (0.15+1.35 mM)	-	11 ± 12%	0.4 ± 0.3 s^{-1}	3.6 ± 1.1 s^{-1}	58	23	504 ±100 ms
GTPγS+GTP (0.5+1 mM)	-	45 ± 16%	0.6 ± 0.2 s^{-1}	10 ± 5 s^{-1}	69	20	825 ± 160 ms

Supp. Table 3| MLE fitting results for $\tau_{unproductive}$. Errors are reported as two standard deviations (95%) confidence intervals of the fits. A single-exponential fit was performed if sufficient. The last column shows sample mean and standard error of the mean (SEM).

$\tau_{unproductive}$

	k_{single}	f_1	k_1	k_2	N event	N ribosome	Mean ± SEM
WT	-	21 ± 18	1 ± 0.8	8.2 ± 3.4	63	14	300 ± 80 ms
H92Q	-	26 ± 56	1.0 ± 1.3	3.3 ± 2.3	29	11	500 ± 120 ms
GDPNP+GTP (0.5+1 mM)	8 ± 3	-	-	-	28	11	125 ± 30 ms
GTPγS+GTP (0.5+1 mM)	-	24 ± 24	3.5 ± 3.4	36 ± 20	25	9	90 ± 30 ms

Supp. Table 4| Evaluation of statistical significance of differences (two-sided) in CDF distributions as compared to those of WT EF-G with GTP. p-values for two-sample Welch's t-tests, Kolmogorov-Smirnov tests (KS tests), and two-sample Mann-Whitney U Tests are shown in the table below. Note that for GTP analog conditions, these statistical tests were run on the entire distribution which includes a subset of normal GTP events.

	Welch p-value	KS p-value	MW p-value
H92Q $\tau_{unwinding}$	0.29	0.99	0.57
GTPγS+GTP $\tau_{unwinding}$ (0.5+1 mM)	0.02	0.10	0.04
GDPNP+GTP $\tau_{unwinding}$ (0.5+1 mM)	0.19	0.77	0.43
H92Q $\tau_{release}$	1.1e-12	2.3e-15	1.3e-16
GTPγS+GTP $\tau_{release}$ (0.5+1 mM)	0.01	0.25	0.39
GDPNP+GTP $\tau_{release}$ (0.5+1 mM)	5.8e-4	1.9e-3	3.3e-5
H92Q $\tau_{unproductive}$	0.17	0.01	0.001
GTPγS+GTP $\tau_{unproductive}$ (0.5+1 mM)	0.02	1.1e-3	1.5e-4
GDPNP+GTP $\tau_{unproductive}$ (0.5+1 mM)	0.06	0.09	0.2

Supp. Table 5| Counts of productive EF-G binding events and number of ribosomes for lower-throughput single-molecule conditions.

	N event	N ribosome
EF-G H92A (hairpin assay)	5	4
L7 V67D (hairpin assay)	6	5
EF-G WT (assisting force assay)	7	6
EF-G + FA (assisting force assay)	8	6
EF-G H92Q (assisting force assay)	7	7
EF-G H92A (assisting force assay)	5	4
EF-G WT + GDPNP + GTP (assisting force assay)	16	13
EF-G WT + GTPγS + GTP (assisting force assay)	10	9
